# Cases Read before the Chicago Medical Society

**Published:** 1864-05

**Authors:** Hiram Wanzer

**Affiliations:** Chicago, Ill.


ARTICLE XXII.

### CASES READ BEFORE THE CHICAGO MEDICAL SOCIETY.

By HIRAM WANZER, M.D., of Chicago, Ill.

Mr. President and Gentlemen:—Permit me to present you a synopsis of a few surgical cases, with a pathological specimen:—

The first case was an injury of the leg. I was called to Capt. B., at Bridgport, on the 12th of June last. While standing on the bow of his boat, his right leg was caught above the ankle, in a coil of running-rope, attached to a tug. It started instantly, producing a compound comminuted fracture of the tibia and fibula at the lower third. Wishing not to shoulder the entire responsibility, I despatched a messenger for Prof. E. Andrews and Dr. A. Fisher. After administering the anæsthetic, a large detached fragment of bone was removed from the crest of the tibia and the fracture reduced. Finding circulation in the anterior and posterior tibial arteries, conservative surgery was adopted. The soft parts were seriously mutilated. For three or four days, hopes were entertained of his recovery. Granulations began to make their appearance. On the fifth night after the accident, *sudden* untoward symptoms followed. He became delirious, tore off his dressings, brought the limb around at right angles, rupturing a large vessel, probably the posterior tibial. Before we arrived, the blood had extravasated throughout the entire tissues of the leg and nearly all the structures of the thigh. There was but little external hemorrhage. His early habits of dissipation had to do with those uninviting symptoms, and they might have been anticipated by treatment, had we not been misled by a denial of them. He died on the morning of the seventh day, having delirium traumaticum, consequent upon the compound comminuted fracture of the tibia and fibula, and undoubtedly a too sudden withdrawal of his accustomed stimuli.

The second case was an injury of the face. I was called to Martin M., drayman, aged 45, on the 21st of July last. He had been kicked by a horse, over the left eye and face, producing a compound comminuted fracture of the left malar and superior maxillary bones. The bones were so displaced, inverted at different angles in the wound, and detached from their connexions, that reposition was impossible, rendering necessary the extraction of the entire malar bone and that portion of the superior maxillary lying external to the infra-orbital canal and a line falling perpendicularly from it—thus carrying off the outer lower third of the anterior third of the orbital cavity, exposing a triangular opening into the antrum of Highmore, measuring nearly an inch in its diameter. For several days the structures about the eye were swollen; the lids discoloured and œdematus; the wound nearly healed by the first intention. The operation was nearly bloodless. I performed it without chloroform.

Seven weeks from the injury, he received a fall, fracturing the right clavicle at its outer third. I was again summoned; I reduced it in the usual way, by a wedge-shaped cushion in the axilla, approximating the elbow to the side by a figure-of-eight bandage.

He made a rapid recovery from both these accidents, with slight deformities. There is a small depression in the face at the cicatrix, and a protuberance upon the clavicle at the seat of fracture, showing there was not perfect coaptation of the fractured bones. There are two slight pathological conditions following both those injuries, which, however, do not interfere with his accustomed pursuits. First; he suffers, by over-exertion, exhaustion and weakness of the muscles of the arm; also, imperfect vision in the injured eye. The amaurotic symptoms have accompanied him since the accident. Nine months after,

Dr. E. L. Holmes made an ophthalmoscopic investigation. This is his own language:—"By simple inspection, I find nothing abnormal in the eye except a marked dilatation and slight irregularity of the pupil. The patient can read, with some difficulty, the large letters of title pages, at the distance of eight inches, and can see the outlines of buildings across the street. The following opthalmoscopic appearances are discovered:—There are two small irregular dark flocculi floating in the vitreous humor. With this exception, the refracting media are transparent. The papilla of the optic nerve is ill-defined, being partially concealed by a yellow-colored deposit, which extends in irregular points beyond its periphery. The vessels of the retina are somewhat atrophied and covered in places with the yellow deposit just mentioned, which exists in patches over the whole concave surface of the eye. A very few of the vessels of the choroid can be seen at the upper and outer part of the eye. No pigment deposits are anywhere present. This absence of pigment is quite unusual in my experience in cases like this. The general appearance of the retina may be compared in some respects to that represented in Jaeger's ophthalmoscopic plates, No. VIII. The amblyopia is undoubtedly a result of inflammation of the retina and choroid, and possibly of the optic nerve, produced by the injury."

The fourth and last case I will present to the Society. I was called, too, early in February last. The boy, aged five years, had received an extensive injury from a circle of iron, weighing 280 ℔s., falling upon the lower third of the leg. Notwithstanding the pulpified condition of the soft parts, as well as the fracture of the tibia and fibula, an attempt was made to save the limb. The foot retained nearly its normal temperature for thirty-six hours. From this period, gangrene occurred in all the superficial structures below the injury. With the assistance of Prof. E. Andrews, amputation was performed at the middle of the leg. The little patient stood the operation well, and reacted finely. In about one week after, a terrible neuralgia occurred in the stump. The symptoms were somewhat similar to those observed in traumatic tetanus; the paroxysms were not continuous; the liberal use of anodynes had but little effect; opiates seemed rather to aggravate the pain, which gradually disappeared under the use of valerian and hyosciamus. The ligatures were allowed to remain six weeks after the amputation. In accordance with the request of the Chicago Medical Society, I made a dissection of the amputated limb, assisted by Dr. JOHN BARTLETT. The structures were so extensively injured that it was impossible to make a perfect dissection. The cutaneous and cellular tissues were pulpified. The muscular structures were extensively lacerated. The muscular portions of the peroneus brevis, the tibialis posticus, and flexor longus policis, were cut. The tendons of the gastrœnemius plantaris and peroneus longus were uninjured; the anterior and posterior tibial arteries were destroyed, with their accompanying veins; the external saphenus was torn across; the internal saphenus was uninjured; the anterior tibial nerve, with the external division of the musculo-cutaneous, were destroyed; the posterior tibial nerve, with the internal branch of the musculo-cutaneous, were entire. The peroneal vessels were lost in the confusion of the parts. There was compound comminuted fracture of the tibia and fibula. That the foot should retain its temperature for so many hours after these injuries, is remarkable.

